# Involvement of White Matter Language Tracts in Glioma: Clinical Implications, Operative Management, and Functional Recovery After Injury

**DOI:** 10.3389/fnins.2022.932478

**Published:** 2022-07-11

**Authors:** Alexander A. Aabedi, Jacob S. Young, Edward F. Chang, Mitchel S. Berger, Shawn L. Hervey-Jumper

**Affiliations:** Department of Neurological Surgery, University of California, San Francisco, San Francisco, CA, United States

**Keywords:** glioma, language mapping, white matter, plasticity, tractography, magnetoencephalography (MEG), repetitive transcranial magnetic stimulation (TMS)

## Abstract

To achieve optimal survival and quality of life outcomes in patients with glioma, the extent of tumor resection must be maximized without causing injury to eloquent structures. Preservation of language function is of particular importance to patients and requires careful mapping to reveal the locations of cortical language hubs and their structural and functional connections. Within this language network, accurate mapping of eloquent white matter tracts is critical, given the high risk of permanent neurological impairment if they are injured during surgery. In this review, we start by describing the clinical implications of gliomas involving white matter language tracts. Next, we highlight the advantages and limitations of methods commonly used to identify these tracts during surgery including structural imaging techniques, functional imaging, non-invasive stimulation, and finally, awake craniotomy. We provide a rationale for combining these complementary techniques as part of a multimodal mapping paradigm to optimize postoperative language outcomes. Next, we review local and long-range adaptations that take place as the language network undergoes remodeling after tumor growth and surgical resection. We discuss the probable cellular mechanisms underlying this plasticity with emphasis on the white matter, which until recently was thought to have a limited role in adults. Finally, we provide an overview of emerging developments in targeting the glioma-neuronal network interface to achieve better disease control and promote recovery after injury.

## Introduction

Increased extent of surgical resection of both low- and high-grade gliomas has been consistently associated with better seizure control, delayed disease progression, and improved overall survival (Sanai and Berger, 2008; Englot et al., 2012; Brown et al., 2016; Molinaro et al., 2020). However, the benefits of maximal resection are limited by the risks of iatrogenic injury to eloquent structures, or those that subserve motor, sensory, language, and cognitive processing and whose injury leads to potentially permanent functional impairment. Indeed, the development of postoperative neurological impairment not only negatively impacts quality of life, but is also associated with worse survival (McGirt et al., 2009; Hervey-Jumper et al., 2015; Hervey-Jumper and Berger, 2016; Rahman et al., 2016; De Witt Hamer et al., 2021; Aabedi et al., 2022b). This balance is especially challenging to achieve when eloquent white matter tracts are involved in the tumor as they are classically thought to be less resilient to injury and possess less plasticity or recovery potential in comparison to eloquent cortex (Ius et al., 2011; Hamer et al., 2013). Furthermore, given the evidence of improved survival with extended resections of non-enhancing disease in addition to the contrast-enhancing disease, the definition of a maximal safe resection is changing and surgeons are pursuing “supratotal” resections when feasible, making this balance between function and resection even more critical (Rossi et al., 2019, 2021; Jackson et al., 2020; Certo et al., 2021). Among the eloquent white matter tracts in the brain, those involved in the production, integration, and comprehension of language are exceedingly consequential given the strong association between language function and patient-reported outcomes (Gabel et al., 2019; Ammanuel et al., 2021; Aabedi et al., 2022a). Thus, an understanding of how best to preserve and support language function is of paramount importance to clinicians involved in the care of patients with dominant-hemisphere perisylvian gliomas. While articles reviewing white matter language pathways and subcortical language mapping techniques in glioma exist (Chang et al., 2015; Young et al., 2021b), an understanding of how these methods intersect in the context of increasing evidence behind glioma-neuron interactions is lacking. In this article, we provide an overview of (1) the clinical implications of gliomas involving white matter language tracts, (2) techniques currently used to preserve these pathways during surgery, (3) the local and cross-regional adaptations that take place in the setting of injury, and (4) emerging developments in the field of neuromodulation and rehabilitation of language function.

## Clinical Implications of White Matter Language Tract Involvement in Glioma

Gliomas are known to preferentially spread along white matter pathways and blood vessels where they induce pathologic demyelination and vasogenic edema. This process is driven in part by tropic NOTCH1–SOX2 signaling between glioma stem cells and the white matter microenvironment (Wang et al., 2019) and is a radiographic and pathologic hallmark of glial neoplasms. This intimate relationship between gliomas and white matter tracts carries significant clinical consequences as glioma invasion into the white matter is associated with worsened cognitive impairment (Liu et al., 2015, 2020), disease recurrence (Bette et al., 2017), and lower survival (Mickevicius et al., 2015). Disruption of language tracts in particular may have prognostic significance. One study in patients with glioblastoma used a voxel-wise survival analysis to demonstrate that regions where contrast-enhancing lesions overlapped with certain white language matter pathways (the inferior fronto-occipital fasciculus and inferior longitudinal fasciculus) were associated with decreased overall and progression-free survival (Mickevicius et al., 2015). However, because the prior analyses were conducted on a univariate basis (i.e., the inherent spatial correlation between voxels of a given lesion mask was not accounted for), relied on an atlas to derive white matter maps (as opposed to performing tractography for each patient), and did not incorporate tumor volume, the sensitivity and specificity of these findings are unclear. Specifically, whether concurrent involvement of other white language matter tracts such as the superior longitudinal fasciculus or arcuate fasciculus, among others, has implications for survival is poorly understood. In terms of quality of life, patients with gliomas have rated language as the single most important functional domain—above motor ability, memory, and problem solving—in influencing their decisions surrounding their care (Gabel et al., 2019). As a result, a significant body of work has been dedicated to the development, implementation, and evaluation of techniques aimed at preserving and restoring language function in patients with gliomas.

## Invasive and Non-Invasive Techniques for Mapping and Preserving Language Function

Several techniques are used in the pre- and intraoperative settings to localize critical white matter structures involved in language function so surgical planning can optimize their likelihood of preservation. Because each technique has its own set of strengths and weaknesses, many centers opt for a multimodal mapping paradigm where several complementary methods are combined and leveraged to facilitate planning (Carrete et al., 2022). Of these techniques, awake intraoperative mapping with direct electrical stimulation (DES) is considered the clinical gold standard, as it provides unparalleled spatial resolution, real-time feedback, and excellent specificity for the language tasks under consideration (Aabedi et al., 2019, 2021a). Other methods are used as adjuncts, or in patients with contraindications as the primary means of language mapping. Here, we provide an overview of mapping strategies ranging from static imaging modalities to non-invasive functional mapping and finally intraoperative language mapping with DES (Table 1). Some of these techniques were specifically designed for subcortical mapping while others have been adapted with minor modifications to identify eloquent white matter structures in addition to the cortex (i.e., DES). Included in this review are anatomic imaging techniques [i.e., diffusion tensor imaging (DTI)] that reveal structural white matter connections, functional imaging techniques [i.e., functional magnetic resonance imaging (fMRI) and magnetoencephalography (MEG)] that show sites correlated with, but not necessarily essential for, language function, and finally, stimulation-based mapping [i.e., transcranial magnetic stimulation (TMS) and direct electrical stimulation] that demonstrate regions that are necessary for language function by interfering with the neural circuitry. Many of these same techniques have been used to study plasticity and the mechanisms of functional recovery after surgery and tumor progression.

**TABLE 1:** Summary of techniques used to map white matter language tracts in patients with glioma.

Method	Advantages	Limitations	Additional notes
Diffusion tensor imaging (DTI)	• Capable of resolving structural white matter tracts, of which several have been implicated in language function and carry high risk of permanent impairment if injured during surgery. • Can be combined with functional imaging modalities to form hybrid structural/functional connectivity maps	• Often requires setting somewhat arbitrary thresholds (i.e., using fractional anisotropy) to generate images • Traditional DTI sequences have difficulty resolving crossing fibers • Anatomic seed placement for fiber tracking is highly user-dependent • Perilesional signal abnormality affects image accuracy	• Demonstrates excellent sensitivity (>95% in some series) for eloquent language sites when direct electrical stimulation is used as the gold standard
Functional magnetic resonance imaging (fMRI)	• Can identify regions of the cortex (and potentially white matter) involved in language processing preoperatively • In patients unable to undergo awake craniotomy or participate in language tasks, can be acquired at rest or during presentation of auditory stimuli	• Cannot distinguish between regions that are simply co-activated during language tasks vs. those that are essential for language function • Liable to spurious results secondary to tumor angiogenesis (i.e., neurovascular uncoupling)	• Concordance with direct electrical stimulation is inconsistent within the literature • Most reliable application is to identify the hemisphere of language dominance preoperatively
Magnetoencephalography (MEG)	• Similar to fMRI in terms of its ability to perform functional mapping with some additional advantages including substantially better temporal resolution and lack of confounding from neurovascular uncoupling	• As with fMRI cannot distinguish between correlational and causal language sites • Spatial resolution, especially for deeper structures, may be limited by the inverse problem.	• Relatively expensive compared to fMRI; few institutions have MEG capability • May be a useful adjunct to direct electrical stimulation by identifying highly-connected hubs that can lead to early language impairment if resected
Transcranial magnetic stimulation (TMS)	• Capable of non-invasively identifying cortical regions that are essential for language function preoperatively • Can be combined with DTI to perform functional seed-based fiber tracking	• There is a lack of standardization for stimulation parameters for language mapping • Small risk of seizures, pain, and discomfort during stimulation	• Has high sensitivity but variable specificity for identifying eloquent language sites compared to direct electrical stimulation
Awake craniotomy with direct electrical stimulation (DES)	• Clinical gold standard, providing direct assessment of the subcortical white matter • Use for language mapping supported by decades of research • Dynamic and real-time; not liable to brain shift after resection is started	• Requires patient participation during surgery with some relative contraindications • Risk of intraoperative seizure	• Small subset of patients still develop persistent impairment after DES, highlighting the need for multimodal mapping • Various mitigation strategies exist for relative contraindications and seizure abortion

### Diffusion Tensor Imaging

Diffusion tensor imaging is a technique based on diffusion-weighted magnetic resonance imaging that measures and computes various parameters related to the movement of water molecules through different tissues in the nervous system (Yang et al., 2011). Because the diffusion of water is more restricted (i.e., anisotropic) along axonal processes compared to cell bodies, DTI can be used to generate three-dimensional structural images of white matter tracts in the brain (Soares et al., 2013). Several of these tracts are imperative for language function by providing the physical infrastructure required for the emergence of networks where the integration of phonological, lexical, semantic, and articulatory information takes place (Chang et al., 2015; Aabedi et al., 2021b). Specifically, a causal role has been established for components of the superior longitudinal fasciculus (SLF), arcuate fasciculus (AF), and inferior fronto-occipital fasciculus (IFOF), whereas, the middle longitudinal fasciculus (MLF) and inferior longitudinal fasciculus (ILF) likely participate in, but may not be essential for, language function. Contemporary “dual stream” models of language processing propose that the IFOF, ILF, and uncinate fasciculus (UF) integrate information within the ventral stream where semantic and syntactic analyses largely take place, while the SLF and AF belong to the dorsal stream where phonological and articulatory processing occur. Data supporting the necessity of the SLF, AF, and IFOF mainly come from stimulation mapping or lesion studies. Stimulation of SLF II and SLF III (but not SLF I) result in impaired motor coordination (Maldonado et al., 2011) or dysarthria with intact comprehension. Furthermore, patients with the non-fluent or agrammatic variant of primary progressive aphasia generally demonstrate intrinsic injury to the SLF, which implies an additional role in the formation of syntax (Wilson et al., 2012). Similarly, lesions to the AF classically lead to conduction aphasia and anomia (Duffau et al., 2002). Finally, stimulation of the IFOF (particularly the anterior component) leads to semantic paraphasias and postoperative impairment if resection is continued in close proximity to the tract (Duffau et al., 2005). The MLF and ILF, on the other hand, while implicated in language processing, may not be required for intact functioning. For instance neither stimulation of nor resection near the MLF leads to naming impairment despite its structural connectivity to critical language cortices such as the superior temporal gyrus (De Witt Hamer et al., 2011). A parallel study interrogating the role of the ILF showed that even partial transection of the tract could be tolerated without postoperative language impairment (Mandonnet et al., 2007). Whether the UF and the frontal aslant tract (FAT) are absolutely essential for language function remains under debate. While one study showed that resection of the UF led to postoperative impairments in the retrieval of proper names (Papagno et al., 2011), another showed that stimulation and resection of components of the tract could be conducted without consequence (Duffau et al., 2009a). Similarly, transection of the FAT has been described as leading to only a transient aphasia as well as supplementary motor area syndrome and therefore may serve primarily in motor planning (Young et al., 2020b,2021a).

In the operating room, DTI can be used with stereotactic neuro-navigation systems to help identify the boundaries of safe resection. Bello et al. (2008) computed the concordance between DTI and DES, the clinical gold standard for functional language mapping, in 64 patients with low- and high-grade glioma and found that DTI had a 97% sensitivity for naming sites. Notably, the tumor grade was found to be correlated with the risk of identifying language tracts proximal to the margins of the tumor: in low-grade gliomas, the white matter tracts were frequently found within the mass and infiltrated by tumor, while in high grades, the white matter tracts were instead near the margins. These findings were subsequently validated in a larger cohort with the caveat that a low fractional anisotropy threshold was required to properly resolve the tracts (Bello et al., 2010). Studies from other groups similarly showed that DTI of the corticospinal tract had at least an 89% sensitivity for DES (Zolal et al., 2012; Javadi et al., 2017) and that language tracts had a sensitivity of 81% (Leclercq et al., 2010). Given the high sensitivity of DTI, and therefore the low likelihood of identifying functional language sites in subcortical regions outside of the reconstructed white matter language tracts, DTI can be combined with DES to tailor stimulation trials and improve the efficiency of the operation. Indeed, Bello et al. (2010) found that the combination of DTI with DES decreased the length of surgery, patient fatigue, and the rate of intraoperative seizures. Nonetheless, despite these strengths, DTI has some limitations including the assumption that fiber tracts are unidirectional (complicating the interpretation of crossing or diverging tracts) as well as a dependence on user-defined thresholds for generating the maps. Moreover, signal abnormalities from the tumor and its associated edema can confound extracted DTI parameters. Some of these limitations have been addressed by more contemporary sequences such as high angular resolution diffusion-weighted imaging (HARDI), at the expense of longer acquisition times (Caverzasi et al., 2016) or by combining DTI with other imaging modalities. However, despite the advantages of HARDI in resolving intersecting tracts that belong to distinct fiber populations, especially in white matter language pathways where this occurs frequently (Kuhnt et al., 2013), studies demonstrating a direct clinical benefit of HARDI over DTI in guiding maximal safe resection of gliomas are lacking.

### Functional Magnetic Resonance Imaging

Functional magnetic resonance imaging is a functional imaging technique that measures voxel-level fluctuations in blood-oxygen-level-dependent (BOLD) signal to identify cognitively and behaviorally relevant regions of the brain (Logothetis, 2008). fMRI has been used during preoperative planning to determine laterality (i.e., the hemisphere predominantly engaged by language), uncover resting-state functional connectivity networks, and identify regions with task-dependent changes in BOLD signal that may be involved in local computational processing. Traditionally, fMRI was only interpreted in the context of gray matter, as many studies regressed out subcortical signals based on the assumption that flow-dependent changes in oxygen delivery were largely absent in the white matter (Bartoň et al., 2019). However, more recent evidence indicates that physiologic signals can indeed arise from the white matter and that white matter tracts can be reconstructed from functional correlation tensors acquired from fMRI (Ding et al., 2016; Grajauskas et al., 2019; Li et al., 2019). Moreover, fMRI can be combined with tractography to provide a functional correlate to anatomic technique, potentially increase the specificity of identifying eloquent white matter language tracts (Sanvito et al., 2020). However, whether the use of combined anatomo-functional modalities leads to clinically meaningful improvements in outcomes requires further study.

Because fMRI has contributed greatly to our understanding of where distinct components of language are processed in the brain and their relative time courses (Matthews et al., 2003), its utility in surgical planning for patients with gliomas involving language areas has been studied extensively. In contrast to DTI, however, the adoption of fMRI in the operating room for language mapping in patients with glioma has been less uniform. This is likely due to inconsistent findings in the literature regarding the concordance between language sites identified *via* fMRI and DES (Roux et al., 2003; Giussani et al., 2010; Kuchcinski et al., 2015; Cochereau et al., 2016). Several criticisms are presented for fMRI which may underly these inconsistencies, including the idea that (1) gliomas induce angiogenesis which can confound local BOLD estimates as a function of their grade and histopathology [i.e., neuro-vascular uncoupling, Pak et al. (2017)], (2) some fMRI activations may be epiphenomenal and therefore not indicative of regions that are essential for language function (Weber and Thompson-Schill, 2010), and (3) substantially different results can be obtained by modifying the pre-processing pipeline and hemodynamic response functions (Tanabe et al., 2002). Nevertheless, fMRI remains an adjunct for determining laterality preoperatively (Anderson et al., 2006), mapping language in conjunction with other modalities (Castellano et al., 2017), and studying adaptive functional reorganization of the brain in response to tumor growth and resection (Deverdun et al., 2020).

### Magnetoencephalography

Magnetoencephalography is a functional imaging technique that measures intrinsic magnetic fields produced by synaptic currents. Similar to fMRI, MEG generates multidimensional neural time series that can be used to measure task-based responses and derive resting-state functional connectivity. A significant advantage of MEG over fMRI is that it does not rely on hemodynamic responses and therefore (1) has substantially higher temporal resolution allowing measurement of high-frequency oscillations of neuronal ensembles and (2) is not liable to confounding by the neuro-vascular uncoupling that can occur from glioma-induced angiogenesis. However, one limitation of using MEG for mapping of neural activity is that it requires addressing the inverse problem. In other words, because the activity of a single source (i.e., a neuronal ensemble) is measured at a distance by a variety of spatially distributed sensors (known as superconducting quantum interference devices, SQUIDs) there are an infinite number of ways to model this activity to achieve the sensor data (Baillet and Garnero, 1997; Sato et al., 2004). Several techniques have been developed to address this problem including beamforming and combining MEG with MRI (i.e., magnetic source imaging, MSI), which have allowed for spatial localization of language function and led to novel insights into its cortical processing (Hild and Nagarajan, 2009; Youssofzadeh et al., 2017). Like fMRI, MEG is less frequently used to map white matter structures. Nevertheless, MEG has been previously combined with tractography to study the contributions of white matter language tracts such as the AF to auditory processing (Berman et al., 2016).

Given some of the advantages of MEG over fMRI in patients with glioma, at some centers, MEG has become the preferred preoperative functional imaging modality. MEG has been used to determine laterality preoperatively (Findlay et al., 2012; Hinkley et al., 2020) and demonstrates good spatial concordance with DES for functional language sites (Frye et al., 2009; Hirata et al., 2010). Moreover, there is evidence that MEG can play a complementary role to DES, especially in the white matter. In patients with dominant-hemisphere low- and high-grade gliomas planned for maximal safe resection, Lee et al. (2020) found that removal of high functional connectivity (HFC) hubs, regions of highly correlated activity identified preoperatively with MEG, led to early postoperative language impairment even though those sites were DES negative. Of note, many of these HFC hubs were found in the subcortical white matter near the margins of the tumor. Collectively, these findings suggest that in order to offer the safest resection possible, several different techniques including structural imaging (i.e., DTI), functional non-invasive imaging (i.e., MEG), and invasive intraoperative mapping (i.e., DES) may need to be combined.

### Transcranial Magnetic Stimulation

Of the mapping methods described thus far, TMS has the unique advantage of non-invasively identifying brain regions that are necessary for (i.e., within the causal pathway of) a given cognitive or behavioral operation. TMS achieves this by emitting pulses of magnetic fields that alter neuronal membrane potentials which can lead to excitation, inhibition, or more complex downstream modulation of neural circuitry as a function of the stimulation parameters and targeted areas (Klomjai et al., 2015). TMS has presented several use cases for mapping of subcortical white matter including improving (1) DTI-based tractography in regions of glioma infiltration (Weiss Lucas et al., 2017), (2) surgical and postoperative motor outcomes (Raffa et al., 2018a), and (3) prognostication and operative risk stratification (Sollmann et al., 2020).

In terms of preoperative language mapping in patients with glioma, TMS was traditionally used to identify eloquent cortex, and several studies support its clinical utility (Haddad et al., 2021). Tarapore et al. (2013) and Picht et al. (2013) found that navigated TMS (nTMS) had at least a 90% sensitivity for DES positive language sites, though with mixed specificity (98 vs. 23.8%, respectively). In patients who were not eligible for awake craniotomy, Raffa et al. (2018b) found that nTMS has a 100% sensitivity and 57% specificity for predicting postoperative language impairment measured by a clinically-validated battery. Subsequent studies demonstrated the feasibility of nTMS specifically for mapping of white matter language pathways. Sollmann et al. (2016) performed DTI fiber tracking from nTMS-positive cortical language seeds and was able to identify a maximum 94% of white matter language tracts including the AF, SLF, ILF, and IFOF. As with earlier studies using DTI in patients with glioma, a low fractional anisotropy threshold (i.e., 25%) was necessary to achieve this result. Using nTMS to functionally-define, as opposed to anatomically-define, seeds for DTI fiber tracking presents several advantages, especially when the functional technique identifies “causal” sites rather than simply correlational language sites (i.e., in the case of nTMS vs. fMRI or MEG). For instance, anatomic seeding is highly operator-dependent (Catani et al., 2002) and is complicated by the tumor’s mass effect and perilesional signal abnormalities. Furthermore, functional seeding with nTMS has better concordance with intraoperative DES compared to other functional imaging techniques such as MEG and fMRI (Sollmann et al., 2016). Limitations of the technique include a small risk of seizure and occasional pain or discomfort during stimulation trials.

### Direct Electrical Stimulation

Awake craniotomy with DES represents an invasive functional mapping strategy where electrical current is directly applied to a brain region of interest in the intraoperative setting, modulating the activity of local neural circuitry and leading to either activation or inhibition of a behavioral response. This method, pioneered in the early 1900s by Foerster and subsequently advanced by Penfield, remains the clinical gold standard for the mapping of motor, somatosensory, and language processing. Evidence supporting the use of DES for language mapping to prevent permanent postoperative language impairment is extensive (Sanai et al., 2008; Duffau et al., 2009b; Garrett et al., 2012; Bu et al., 2021) and has facilitated the resection of gliomas involving traditionally “inaccessible” areas such as the insular cortex (Sanai et al., 2010; Hervey-Jumper et al., 2016; Safaee et al., 2016; Hervey-Jumper and Berger, 2019). DES is also capable of capturing the vast inter- and intra-patient variability in the spatial distribution of eloquent language sites and is not liable to inaccuracy in stereotactic navigation secondary to brain shift once resection is initiated (Southwell et al., 2016). Although DES was historically used for mapping of eloquent cortex, strategies for subcortical language mapping emerged soon after, and for a substantial period of time, represented the only method of reliably identifying eloquent white matter structures (Duffau et al., 2002, 2003b).

**FIGURE 1:**
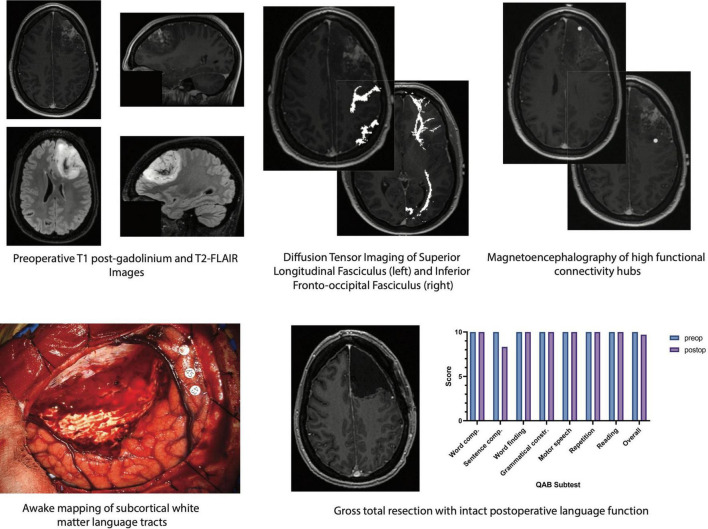
Schematic of multimodal mapping paradigm used to localize and preserve critical white matter structures involved in language function in a patient with a dominant-hemisphere high-grade glioma. (*Top, from left to right*) preoperative imaging demonstrates an expansile contrast-enhancing mass involving the left superior and middle frontal gyri with non-contrast-enhancing signal extending into the subcortical white matter. Diffusion tensor imaging with anatomic seeding reveals several white matter language tracts in close proximity to the tumor including the superior longitudinal fasciculus. The inferior fronto-occipital fasciculus is located deep to the tumor and may be encountered if supratotal resection is pursued. Magnetoencephalography/magnetic source imaging, which can identify regions of high functional connectivity by measuring alpha-band imaginary coherence, shows a hub in the posterior margin of the tumor. (*Bottom, from left to right*) eloquent white matter language tracts are identified in real time *via* awake craniotomy with direct electrical stimulation. Gross total resection of the contrast-enhancing lesion is achieved while leaving structural and functional connections intact, enabling preserved language function even by 2 days postoperative follow-up. Word comp., word comprehension; Sentence comp., sentence comprehension; Grammatical constr., grammatical construction; QAB, quick aphasia battery.

A variety of intraoperative language tasks can be used and tailored to the specific language tracts under investigation (Morshed et al., 2020; Young et al., 2021b). This general procedure involves performing tractography in the preoperative setting and registering the subsequent three-dimensional images with a frameless stereotactic neuro-navigation system, allowing for real-time evaluation of potentially eloquent tracts. As the resection nears a white matter pathway, a bipolar electrode is applied directly to the underlying tissue and biphasic square-waves are generated at 60 Hz, starting at a current amplitude of 1.5–2.0 mA (Duffau et al., 2008). If the site is deemed as DES negative (i.e., does not lead to a language error in at least two of three stimulation trials), resection can be continued safely for another few millimeters until DES is resumed. This method has been used to preserve a variety of language functions including (1) naming in the AF, SLF, IFOF, and UF, (2) repetition in the AF and IFOF, and (3) reading in the ILF (Young et al., 2021b). A schematic demonstrating a multimodal mapping paradigm wherein structural and functional imaging techniques are used to guide DES and preserve language function in a patient with dominant-hemisphere high-grade glioma is depicted in Figure 1.

Direct electrical stimulation may also be combined with adjuncts such 5-aminolevulinic acid (5-ALA) and stimulated Raman histology (SRH) to inform oncologically-derived resection margins. For instance, the use of 5-ALA, an amino acid metabolite that is taken up by glioma cells and converted to a fluorescent product that can be visualized intraoperatively, is associated with significantly increased extents of resection without an increased risk of postoperative neurological impairment (Hadjipanayis et al., 2015). Similarly, SRH can be exploited to identify the true margins of the tumors (i.e., transitions to areas that are free of microscopic infiltrative disease) where further resection may no longer be of oncological benefit to the patient (Pekmezci et al., 2021). Future work on leveraging these techniques to optimally identify functional and oncological margins of safe resection is needed.

Although DES is an essential technique for subcortical language mapping, it has some manageable limitations (Hervey-Jumper et al., 2015). One major intraoperative complication of DES is a stimulation-induced seizure, which can be monitored in real time by electrocorticography and suppressed with iced Lactated Ringer’s solution on the cortex or a propofol infusion if necessary. Relative contraindications to DES such as obesity and chronic obstructive pulmonary disease can be mitigated with advanced airway techniques and cough suppressants, respectively. Finally, concerns surrounding patient anxiety and participation can be addressed with low-dose infusions of anxiolytics, objective wakefulness examinations prior to language mapping, and staged procedures with multimodal mapping (Aabedi et al., 2019).

## Local and Cross-Regional Adaptations After Injury

Despite the use of multimodal mapping including stereotactic navigation with anatomical and functional images and awake craniotomy with DES, postoperative language impairment is still experienced by a small subset of patients. In their landmark study of 250 consecutive patients with dominant-hemisphere perisylvian gliomas undergoing awake craniotomy (Sanai et al., 2008), found that 56 patients had either worsened language function or new impairments 1 week after surgery. However, 6 months later, only four of the surviving 243 patients had a language impairment. In other cases, growth or recurrence of the glioma itself can lead to progressive deficit. However, when patients with recurrent disease undergo repeat awake craniotomy, stimulation mapping has revealed an adaptive displacement of cortical nodes of the language network to regions remote to the original tumor nidus (Southwell et al., 2016). Both of these scenarios highlight the intrinsic plasticity of the language network and have inspired research into the local and cross-regional adaptations that occur as injury from the tumor and surgery interact with persisting functional networks and the glial microenvironment. The mechanisms underlying this plasticity are an area of active investigation as they may carry insights into promoting and accelerating functional recovery for patients with postoperative impairment.

### Clinical Evidence of Plasticity and Underlying Network Mechanisms

Evidence of plasticity in gliomas came from early work demonstrating functional recovery even after radical resection of eloquent areas (Duffau et al., 2003a). For example, removal of the supplementary motor area (SMA) in patients with glioma leads to hemi-akinesia and mutism (i.e., an SMA syndrome) in the immediate postoperative setting. However, by 6 months, a vast majority of patients (exceeding 90% in some series) experience complete neurological recovery (Young et al., 2021a). Recovery of language function after glioma resection in the dominant perisylvian areas is also common and several competing theories as to how this occurs exist in the literature (Krishna et al., 2021b).

One prevailing theory is that the contralateral hemisphere compensates for the injury by developing new (or strengthening existing) functional networks (Duffau, 2006). For instance, Traut et al. (2019) conducted whole-brain MEG in 73 patients with glioma at diagnosis and again after surgery at the point of recurrence. They found a median 29% change in the laterality index (a quantitative measure of the left hemisphere’s dominance in language processing), indicating a shift in global language function toward the contralateral hemisphere. Interestingly, they also found that patients with the highest laterality preoperatively (i.e., biased processing in a single hemisphere) had the largest changes in laterality at recurrence, which suggests the formation of entirely new functional networks. A complete shift in laterality occurred in 5.5% of patients.

Moreover, the development of a glioma itself can induce this contralateral compensation even without surgical resection. For example, Krieg et al. (2013) computed hemispheric dominance ratios (conceptually equivalent to the laterality index) in 15 patients with left-sided gliomas and compared them to 50 healthy controls *via* repetitive nTMS. Using this technique, they found that patients with glioma had significantly higher participation of their contralateral hemisphere in language tasks compared to controls. Further, Jehna et al. (2017) found that increased connectivity of the contralateral white matter language tracts was functionally protective (i.e., associated with fewer language impairments preoperatively). Changes in language network connectivity in non-opercular regions have also been observed. For instance, using resting state fMRI, Fang et al. (2021) found networks alterations involving the contralateral insular cortex in patients with low-grade glioma.

The relative contributions of ipsi- vs. contra-lesional hemisphere in network remodeling remains controversial. For instance, Lizarazu et al. (2020) conducted MEG in patients with low-grade glioma involving language areas at three time points: (1) prior to surgery, (2) 3 months postoperatively, and (3) 6 months postoperatively. In doing so, they found significantly increased alpha-band imaginary coherence (a measure of functional connectivity) in peritumoral regions between preoperative and 3- and 6-month postoperative measurements. Contra-tumoral imaginary coherence values, on the other hand, remained stable across all three recording sessions. Notably, only 12 participants were included and correlations with language endpoints were not provided, limiting clinical interpretation of these findings. Similarly, in a cohort of 14 patients with left-hemispheric low-grade glioma in language areas, Kristo et al. (2015) were unable to detect contra-tumoral increases in functional connectivity. Increases in peri-tumoral activity were noted in postoperative recordings but attributed to brain shift. More recent studies support the notion that both ipsi- and contra-lesional network alterations can support restoration of function after surgery and that the relative contributions of each vary from patient to patient (Yuan et al., 2022). These changes may be more related to functional, as opposed to anatomical, distance from the tumor (Nenning et al., 2020). Finally, there is evidence that regions outside of the cerebral cortex (i.e., the cerebellum) participate in reorganization, providing a more nuanced view of plasticity-related changes to the language network in glioma (Zhang et al., 2018).

This framework for plasticity is supported by shifts in our understanding of how information is organized and processed in the brain. The classical locationist models, which posit that all behaviors and cognitive operations can be localized to circumscribed regions of the cortex, essentially preclude the existence of plasticity (Duffau, 2014). More contemporary network theories on the other hand, which propose that information is processed by cortical hubs or nodes that form dynamic connections when neuronal ensembles briefly synchronize their activities, provide a plausible mechanism (Varela et al., 2001; Matthews et al., 2003). According to this theory, if a given functional network is well-distributed and not reliant on a small number of highly-connected hubs, it can successfully undergo reorganization in the setting of injury, akin to airlines that can accommodate unexpected airport closures by rearranging connecting flights (Bullmore and Sporns, 2012). A major caveat to this theory is that for plasticity to take place, the physical infrastructure supporting the connection of these hubs (i.e., the white matter) must be intact. Indeed, this notion is supported by empirical evidence demonstrating that injury to the subcortical white matter is poorly tolerated and often leads to permanent neurological impairment (Ius et al., 2011).

Although originally thought to be a bystander to cortical plasticity, recent evidence indicates that the white matter plays a key role in promoting it. For instance, in epilepsy, resection of seizure onset zones led to increased contralateral fiber connectivity as measured by DTI, which was associated with improved cognitive functioning (Jeong et al., 2016). Similarly, after resection of arteriovenous malformation, patients experienced a significant increase in the number of fibers within the right hemispheric AF with concurrent improvements in language function (Jiao et al., 2020). Finally, in a longitudinal study of patients with left-hemispheric gliomas, structural changes in SLF laterality (i.e., a shift in fiber tract strength toward the contralateral hemisphere) was correlated with increased contralateral cortical language processing (Amoruso et al., 2020). These findings suggest that changes in structural and functional connectivity act in concert to promote cortical hub relocation toward uninjured cortex and restore some degree of functioning in patients.

### Cellular Mechanisms of White Matter Plasticity

Despite clear evidence supporting white matter changes after injury, the cellular mechanisms underlying these adaptations in patients with glioma remain elusive. Thus, until recently (Krishna et al., 2021b) studies of plasticity at the cellular level have been largely limited to models of embryological development, acute ischemic stroke, and trauma. In the spinal cord, axonal sprouting in response to peptide factors such as neutrophin-3 (NT-3) has been observed (Grill et al., 1997; GrandPré et al., 2002). Such neurotrophic factors including NT-3, nerve growth factor (NGF), and brain-derived neurotrophic factor (BDNF) were originally identified in studies of normal embryological development of the corticospinal tract and peripheral nervous system, but now appear to play a role in adults by promoting the regeneration of axons after injury (Schnell et al., 1994). Interestingly, axonal regeneration can be enhanced by relative increases in activity (either endogenously or exogenously) at the level of the neuronal cell body (McGregor and English, 2019). For instance, electrical stimulation has been shown to increase the likelihood of successful re-innervation of muscles after acute axonal injury to motor neurons. It is hypothesized that stimulation mimics the normal backpropagating calcium wave that occurs after injury (Wolf et al., 2001) and promotes recovery by increasing the expression of neurotrophic factors (i.e., NGF) by neurons (i.e., dorsal root ganglia and motor neurons) and glia (i.e., Schwann cells) bi-directionally. Parallel work in the brain has demonstrated ongoing adult neurogenesis (including axonal sprouting) in response to excitation (so-called “excitation-neurogenesis coupling”) (Deisseroth et al., 2004) which, in models of acute stroke, has been shown to be mediated by the release of BDNF (Schäbitz et al., 2007). As further evidence, single nucleotide polymorphisms in the gene coding for BDNF have been shown to modulate the likelihood of a clinically relevant response to rTMS: in acute ischemic stroke, patients with a Val/Val polymorphism had greater recovery of motor function after a 10-day course of rTMS compared to those with a Val/Met polymorphism (Chang et al., 2014). Such studies provide plausible cellular mechanisms for functional recovery in response to neuromodulation in humans, though more work is required to establish a causal link.

Increases in white matter volumes and the necessary support of new axons may be achieved in part by the proliferation of glial cells (Walhovd et al., 2014). For instance, oligodendrocyte precursor cells can differentiate and myelinate axons in the adult brain (Rivers et al., 2008) and form synapses with neurons (Bergles and Richardson, 2016). This can lead to changes in several axonal properties including diameter, internode length, and myelination, all of which affect conduction speed and the formation of functional networks (Nunez et al., 2015; Sampaio-Baptista and Johansen-Berg, 2017). Similar to post-injury adult neurogenesis and axonal regeneration, glial proliferation is also mediated by neurotrophic factors. For instance, in a mammalian model of ischemic stroke, exogenous administration of BDNF led to an increase in oligodendrocyte precursors which, in turn, resulted in (1) improved structural connectivity (measured by DTI) and (2) better motor recovery compared to controls (Ramos-Cejudo et al., 2015). Conversely, knockout of NT-3 leads to decreases in astrocyte, microglia, and oligodendrocyte populations (Kahn et al., 1999).

In glioma, however, the use of exogenous neurotrophins or treatments that increase their endogenous levels to promote functional recovery presents a largely unexplored clinical quandary. Indeed, many of the same paracrine factors that are implicated in the repair and recovery processes that take place after injury are also involved in the proliferation of glioma cells and progression of disease (Gillespie and Monje, 2018). For instance, application of conditioned media containing BDNF to glioma cell cultures leads proliferation of glioma cells, which is effectively suppressed through inhibition of TRKB (the receptor for BDNF) (Venkatesh et al., 2015). Furthermore, *in vivo* optogenetic stimulation of neural circuits embedded with patient-derived glioblastoma xenograft leads to tumor growth, a process that may be mediated by increases in BDNF and neuroligin-3 (Pan et al., 2021).

## Future Directions—Impacting Disease Trajectory and Promoting Postoperative Recovery Through Neuromodulation

Recent evidence demonstrating that gliomas integrate within functional neuronal networks and proliferate in response to synaptic activity carries significant clinical and therapeutic implications for disease control and post-treatment recovery (Venkatesh et al., 2019; Young et al., 2020a; Aabedi et al., 2021c; Krishna et al., 2021a). For instance, hyper-excitable networks can be targeted with stimulation or other forms of neuromodulation, which may have an impact on tumor progression, though work in this arena is still in its infancy (Sprugnoli et al., 2021a,b). Clinical trials of non-invasive high-frequency electrical field application (i.e., tumor treating fields) to glioblastoma has been met with some success, though whether this treatment has effects beyond purely anti-mitotic activity is unclear (Carrieri et al., 2020). Neuromodulation also offers exciting applications in terms of promoting recovery. In a randomized, double-blinded trial, patients with glioma who presented with post-operative hemiparesis due to subcortical ischemia underwent low-frequency TMS or sham stimulation for seven consecutive days prior to physical therapy (Ille et al., 2021). Compared to sham, TMS led to significant improvements in motor function across two validated scales, with an impressive number needed to treat of 2.2 to achieve the minimum clinically important difference. In the stroke literature, analogous stimulation protocols in patients with post-stroke aphasia led to significant changes in white matter structural connectivity across the left hemisphere (Allendorfer et al., 2012). Interestingly, inhibitory TMS to the contralateral hemisphere has led to an immediate improvement in language function (Turkeltaub et al., 2012). Whether similar changes in the white matter underly post-stimulation improvements in neurologic function and the role of interhemispheric inhibition in glioma is unknown. Interventional studies on improving language function in patients with glioma and promoting recovery after surgery or other adjuvant treatments are lacking. However, existing work in the stroke literature as well as more recent work in accelerating motor recovery in glioma provide promising avenues for future trials.

## Conclusion

The management of gliomas involving white matter language tracts is challenging, but advances in mapping techniques over the last several decades have made resection of these lesions safer. A multimodal mapping paradigm wherein structural connectivity is evaluated with diffusion-based imaging and combined with functional techniques such as MEG or TMS can be used to tailor subcortical direct electrical stimulation trials during awake craniotomy and improve the efficiency and safety of the operation. Language impairment as a result of disease progression, treatment, or a combination of the two is common and threatens both quality of life and survival in patients with glioma. Local and long-range network plasticity provides a mechanism for recovery of language function and may be intervened upon *via* neuromodulation to accelerate the process.

## Author Contributions

All authors listed have made a substantial, direct, and intellectual contribution to the work, and approved it for publication.

## Conflict of Interest

The authors declare that the research was conducted in the absence of any commercial or financial relationships that could be construed as a potential conflict of interest.

## Publisher’s Note

All claims expressed in this article are solely those of the authors and do not necessarily represent those of their affiliated organizations, or those of the publisher, the editors and the reviewers. Any product that may be evaluated in this article, or claim that may be made by its manufacturer, is not guaranteed or endorsed by the publisher.
